# Modeling Aberrant Angiogenesis in Arteriovenous Malformations Using Endothelial Cells and Organoids for Pharmacological Treatment

**DOI:** 10.3390/cells14141081

**Published:** 2025-07-15

**Authors:** Eun Jung Oh, Hyun Mi Kim, Suin Kwak, Ho Yun Chung

**Affiliations:** 1 Department of Plastic and Reconstructive Surgery, Cell and Matrix Research Institute, School of Medicine, Kyungpook National University, Kyungpook National University Hospital, Daegu 41944, Republic of Korea; fullrest74@knu.ac.kr (E.J.O.); suin8349@knu.ac.kr (S.K.); 2 BK21 FOUR KNU Convergence Educational Program of Biomedical Science for Creative Future Talents, School of Medicine, Kyungpook National University, Daegu 41944, Republic of Korea; sarang7939@knu.ac.kr

**Keywords:** thalidomide, arteriovenous malformation, endothelial cells, organoids

## Abstract

Arteriovenous malformations (AVMs) are congenital vascular anomalies defined by abnormal direct connections between arteries and veins due to their complex structure or endovascular approaches. Pharmacological strategies targeting the underlying molecular mechanisms are thus gaining increasing attention in an effort to determine the mechanism involved in AVM regulation. In this study, we examined 30 human tissue samples, comprising 10 vascular samples, 10 human fibroblasts derived from AVM tissue, and 10 vascular samples derived from healthy individuals. The pharmacological agents thalidomide, U0126, and rapamycin were applied to the isolated endothelial cells (ECs). The pharmacological treatments reduced the proliferation of AVM ECs and downregulated miR-135b-5p, a biomarker associated with AVMs. The expression levels of angiogenesis-related genes, including *VEGF*, *ANG2*, *FSTL1*, and *MARCKS*, decreased; in comparison, *CSPG4*, a gene related to capillary networks, was upregulated. Following analysis of these findings, skin samples from 10 AVM patients were reprogrammed into induced pluripotent stem cells (iPSCs) to generate AVM blood vessel organoids. Treatment of these AVM blood vessel organoids with thalidomide, U0126, and rapamycin resulted in a reduction in the expression of the EC markers CD31 and α-SMA. The establishment of AVM blood vessel organoids offers a physiologically relevant in vitro model for disease characterization and drug screening. The authors of future studies should aim to refine this model using advanced techniques, such as microfluidic systems, to more efficiently replicate AVMs’ pathology and support the development of personalized therapies.

## 1. Introduction

Arteriovenous malformations (AVMs) are congenital vascular anomalies characterized by an abnormal, direct connection between arteries and veins that bypasses the capillary networks. This aberrant vascular architecture leads to various clinical manifestations, including pain, pulsation, and local hyperthermia, depending on the size and location of the malformation. Notably, AVMs most commonly affect the head and neck region, followed by the extremities and other areas [1].

The management of AVMs is challenging due to their complex vascular anatomy and the potential for serious complications such as ischemic skin ulceration, bleeding, bone destruction, and even cardiac failure. Traditional treatment modalities include surgical resection and endovascular embolization; however, these approaches can be invasive and may be unsuitable for all patients, especially those with extensive or anatomically challenging lesions. Consequently, there is increasing interest in pharmacological therapies that target the molecular and cellular mechanisms underlying AVM pathology [2,3].

Several pharmacological agents have been investigated to determine their potential to modulate the progression of AVMs. Indeed, sirolimus (rapamycin), an mTOR inhibitor, has been shown to reduce vessel density and promote vascular normalization in AVM models by inhibiting the proliferation and angiogenic signaling of endothelial cells (ECs). However, the clinical efficacy of sirolimus in treating AVMs remains to be elucidated. Moreover, while sirolimus has shown therapeutic benefit in some patients by reducing lesion progression and alleviating symptoms, the overall clinical response remains variable, and its effectiveness is not uniformly established [4]. In addition, among known MEK inhibitors, U0126 has been widely used in experimental studies to block ERK1/2 activation and investigate the downstream effects of inhibiting the MAPK/ERK signaling pathway in vascular ECs [5]. U0126 has also been investigated to determine its potential in treating AVMs. Moreover, the results of preclinical studies have shown that MEK inhibition can suppress the progression of AVMs, particularly those associated with somatic activation mutations in MAP2K1, which encodes MEK1. Despite these promising findings, clinical evidence remains limited, and the precise mechanisms through which MEK inhibitors exert their therapeutic effects on AVM ECs are not fully understood [6,7].

The results of recent studies demonstrate that thalidomide effectively hinders angiogenesis and reduces the size of vascular lesions in AVM models, thereby supporting its potential as a repurposed therapeutic agent for AVMs [8]. Thalidomide is widely recognized for its anti-angiogenic and immunomodulatory properties, primarily through its interaction with endothelial cells. One of the major mechanisms by which thalidomide exerts its anti-angiogenic effects is the suppression of vascular endothelial growth factor (VEGF), leading to the inhibition of new blood vessel formation and the suppression of tumor growth and inflammation [9]. In addition, thalidomide reduces the production of inflammatory cytokines, notably tumor necrosis factor alpha (TNF-α), thereby dampening endothelial activation and chronic inflammation. These effects are mediated in part through the inhibition of the NK-κB signaling pathway, which plays a critical role in the survival and immune regulation of ECs [10]. Nonetheless, despite these therapeutic benefits, thalidomide is associated with teratogenic effects when administered during pregnancy, as it disrupts embryonic vasculature and causes congenital malformations. Therefore, the clinical use of thalidomide requires strict regulation and monitoring to minimize risk [11].

Given the complexity of AVMs, the development of reliable disease models has become essential for understanding their pathophysiology and evaluating therapeutic candidates. Thus, various AVM models have been established to replicate key features of the disease in both biological and physiological contexts. In vivo animal models, such as genetically engineered mice, have demonstrated utility in evaluating the efficacy of anti-angiogenic drugs in a whole-organism context [12]. Furthermore, ex vivo systems, including isolated vessel cultures from AVM tissues, enable a clearer examination of cell-specific responses and signaling dynamics under controlled environments [13]. Recent developments in microfluidic-based AVM-on-a-chip platforms have enabled high-throughput drug screening and the replication of abnormal hemodynamics and endothelial behaviors of AVMs in reproducible, miniaturized environments [14]. The combination of these models offers platforms for preclinical drug testing and contributes to a deeper understanding of AVM biology and potential therapeutic mechanisms.

Therefore, in this study, we aimed to investigate the therapeutic potential of pharmacological agents, including thalidomide, U0126, and rapamycin, on AVM ECs. These drugs, known for their anti-angiogenic or signaling inhibitory properties, were evaluated to determine their ability to preserve the pathological features of the vasculature in AVMs. Hence, by treating patient-derived AVM ECs with pharmacological agents, we assessed their effects on cellular behavior, including proliferation, angiogenesis, and gene expression, to identify potential therapeutic mechanisms. Furthermore, we compared the phenotypic and molecular characteristics of AVM ECs with those of normal ECs to identify disease-specific alterations and therapeutic targets. To complement conventional cell-based analyses, we also incorporated an AVM organoid model to assess drug responses in a more physiologically relevant context. Through this integrated approach, we aimed to provide foundational insights into the pharmacological modulation of AVMs and propose a screening strategy that may inform the development of less invasive, targeted treatments for AVM patients.

## 2. Materials and Methods

### 2.1. Patient-Derived Human Samples and Cell Isolation

**Figure 1:**
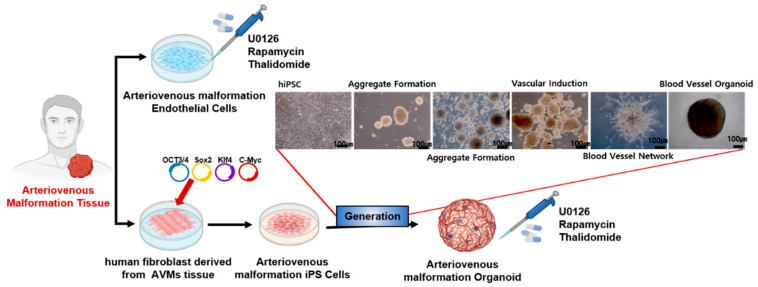
Generation of AVM organoids and analysis of response to pharmacological treatment. The generation and analysis of AVM organoids derived from human fibroblasts obtained from AVM tissue. Human dermal fibroblasts were reprogrammed into induced pluripotent stem cells (iPSCs), followed by differentiation, during which organoid structures were formed, mimicking the vascular architecture associated with AVMs. The AVM ECs and AVM organoids were treated with pharmacological inhibitors (thalidomide, rapamycin, and U0126) to assess drug responses.

Building upon our previous work in establishing a vascular organoid platform using patient-derived ECs and fibroblasts [15], in this study, we expanded the sample to include 30 different human samples. This decision enabled the pharmacological evaluation of AVMs. In more detail, ECs were isolated from 10 vessel samples from patients with AVMs and 10 vessel samples from the tissue of healthy individuals. In addition, 10 skin samples were obtained from the tissues of patients with AVMs for hiPSC reprogramming (Figure 1).

All samples were obtained during surgeries conducted at the Vascular Anomalies Center of a tertiary general hospital. To ensure consistency and disease specificity, visceral organ tissues (e.g., brain, gastrointestinal, and pulmonary tissues) and syndromic AVM (e.g., Parkes Weber syndrome, capillary malformation, arteriovenous malformation, and hereditary hemorrhagic telangiectasia) were excluded. Immediately after resection, the tissues were immersed in phosphate-buffered solution (PBS, pH 7.4) and transferred to the laboratory. The study was conducted in accordance with the Declaration of Helsinki and approved by the institutional review board of Kyungpook National University Hospital (Approval no. KNUH 2023-04-004-002). Informed consent was obtained from all patients (Table 1).

### 2.2. Pharmacological Treatment of AVM Endothelial Cells and AVM Organoids

The experimental concentrations of thalidomide, rapamycin, and U0126 used in the AVM ECs and AVM organoid treatment were as follows: thalidomide (10 μM, T44; Sigma-Aldrich, St. Louis, MO, USA), rapamycin (10 nM, R8781; Sigma-Aldrich, St. Louis, MO, USA), and U0126 (10 μM, #9903; Cell Signaling Technology, Danvers, MA, USA). Each compound was prepared according to standard dilution protocols, and cells were treated for 24 h with a final dimethyl sulfoxide (DMSO) concentration of 0.1%. All conditions were maintained to ensure consistency.

### 2.3. Phenotypic and Molecular Characterization of AVM ECs

#### 2.3.1. Isolation and Culture of Human Endothelial Cells (hECs)

The isolation of ECs followed previously published methods with minor modifications [15]. Briefly, AVM tissues were rinsed with PBS (LB004-02, Welgene, Gyeongsan, Republic of Korea), cut into fragments, and incubated in Dispase II (Gibco^TM^, 17105-041, Thermo Fisher, Waltham, MA, USA) at 4 °C for 24 h. Following incubation, the dermal layer was collected and digested with collagenase type I (4196, Worthington, OH, USA) at 37 °C and 170 rpm for 1 h. The resulting suspension was passed through a 70 μm nylon filter and centrifuged at 1000 rpm for 5 min. The cells were seeded in EMB-2 media (cc-3156, Lonza, Basel, Switzerland) and maintained at 37 °C with 5% CO_2_.

#### 2.3.2. Immunofluorescence and Brightfield Imaging Analysis

Brightfield images were acquired using an inverted microscope (Axio, Carl Zeiss, Jena, Germany) with an objective lens.

To perform immunofluorescence staining, the cells were fixed in 4% paraformaldehyde on a rocking platform at 25 °C for 1 h, permeabilized with 0.1% Triton™ X-100 (T9284, Sigma-Aldrich, St. Louis, MO, USA), and blocked with 0.1% Tween^®^ 20 (P1379, Sigma-Aldrich, St. Louis, MO, USA). Primary antibodies ANG2 (ab153934, Abcam, Cambridge, UK), VEGF (ab52917, Abcam, Cambridge, UK), and CD31 (ab76533, Abcam, Cambridge, UK) were subsequently applied overnight (≥16 h) at 4 °C. The secondary antibody (goat anti-rabbit IgG H&L Alexa Fluor^®^ 488 A-11008, Invitrogen, Waltham, MA, USA) was applied for 3 h at room temperature (RT) in the dark. Nuclei were counterstained with DAPI and Hoechst nucleic acid stains (H3570, Invitrogen, Waltham, MA, USA) for 10 min. The Vectashield Antifade Mounting Medium (H-1000, Vector Laboratories, Newark, CA, USA) was used. Immunofluorescence imaging was performed using a Leica STED (STeLLARIS) 5 confocal microscope (Leica, Wetzlar, Germany).

#### 2.3.3. Quantitative Real-Time Polymerase Chain Reaction (qRT-PCR)

Total RNA was extracted using Trizol^TM^ Reagent (15596026, Thermo Fisher Scientific, Waltham, MA, USA), and the RNA concentration and purity were assessed using a NanoDrop spectrophotometer (DS-11, DeNovix, Wilmington, DE, USA), with 260/280 ratios between 1.8 and 2.0 indicating acceptable purity. Reverse transcription into cDNA was performed using RT Premix (EBT-1515, ELPIS, Gwangju, Republic of Korea). Next, qRT-PCR was performed using SYBR Green Supermix reagent (Bio-Rad, Hercules, CA, USA) on a CFX96 system. GAPDH was used as a control to quantify the target gene. Gene expressions were analyzed using the 2^−ΔΔCT^ method (Table 2).

#### 2.3.4. TaqMan Assay (Mir)

All experiments were performed in triplicate and normalized to the endogenous control (has-miR-361-5p; Assay ID: 478056_mir, Thermo Fisher Scientific, Waltham, MA, USA). The expression of hsa-miR-135b-5p (Assay ID: 478582_mir, Thermo Fisher Scientific, Waltham, MA, USA) was quantified using TaqMan Advanced MicroRNA Assays (Applied Biosystems, Foster City, CA, USA) and analyzed using the 2^−ΔΔCT^ method (Table 3).

#### 2.3.5. Tube Formation Assay

For the angiogenesis analysis, an extracellular matrix solution (angiogenesis assay kit, in vitro, ab204726, Abcam, Cambridge, UK) was prepared at 37 °C for 30 min and then observed under a fluorescence microscope (AXIO, Carl Zeiss, Oberkochen, Germany). During the analysis, the total vessel lengths and number of junctions were measured in the field (n = 3) using Fiji ImageJ software 1.54h (National Institutes of Health, Bethesda, MD, USA).

### 2.4. Establishment and Immunostaining of AVM Organoids

#### 2.4.1. Isolation and Reprogramming of Human Fibroblasts

The isolation and reprogramming of human fibroblasts were performed following a previously described method [15]. Dermal tissues were treated with Dispase II (Gibco^TM^, 17105-041, Thermo Fisher, Waltham, MA, USA) overnight and then digested with collagenase type II and filtered through a 70 µm cell strainer (Corning^®^, Corning, NY, USA) using Dulbecco’s Modified Eagle Medium (DMEM/HIGH; HyClone, Logan, UT, USA). The human fibroblasts were cultured to ~80% confluency prior to transfection with non-modified RNA (NM-RNA) using the StemRNA™ 3rd Gen Reprogramming kit (Cat. No. 00-0076, Stemgent^®^, Reprocell Inc., Tokyo, Japan). The resulting hiPSC colonies were maintained on a Vitronectin (VTN-N) (A31804, Gibco, Thermo Fisher Scientific, Waltham, MA, USA)-coated dish containing TeSR^TM^-E8^TM^ medium (05990, Stemcell Technologies, Vancouver, BC, Canada).

#### 2.4.2. Generation of Blood Vessel Organoids (BVOs)

hiPSCs were differentiated into vascular organoids using the STEMdiff™ Blood Vessel Organoid kit (#100-0651, Stemcell Technologies, Vancouver, BC, Canada), following the manufacturer’s protocol. The aggregates were embedded in a collagen/Matrigel^®^ matrix and cultured sequentially in mesoderm induction, vascular induction, and maturation media. On day 11, the BVOs were isolated and transferred to 96-well ultra-low attachment plates (MS-9096UZ, Sumitomo Bakelite, Tokyo, Japan) for further analysis [14].

#### 2.4.3. Whole-Mount Immunofluorescence of AVM Organoids

The organoids were fixed in 4% paraformaldehyde and permeabilized using a methanol gradient (50%, 80%, and 100%). After blocking, the samples were stained with CD31 (ab76533, Abcam, Cambridge, UK) and the secondary antibody (goat anti-rabbit IgG H&L Alexa Fluor^®^ 488 A-11008, Invitrogen, Waltham, MA, USA). Nuclear counterstaining was performed using DAPI and Hoechst (H3570, Invitrogen, USA). The BVOs were cleared using RapiClear^®^ 1.47 (RC147001, SUNJin Lab, Hsinchu City, Taiwan) and imaged using a Leica STELLARIS5 confocal microscope (Leica, Wetzlar, Germany).

### 2.5. Statistical Analysis

All experiments were conducted using biological replicates from 10 individuals. The data are presented as the mean ± standard deviation (SD) of three technical replicates of the experiment. Statistical analysis was performed using GraphPad Prism 10.5.0 (GraphPad Software, San Diego, CA, USA). Differences between groups were evaluated using one-way analysis of variance (ANOVA) followed by Dunnett’s post hoc test, with each experimental group compared to the control. Statistical significance was defined as a *p*-value ≤ 0.05.

## 3. Results

### 3.1. Pharmacological Treatment of AVM ECs

#### 3.1.1. Morphological Changes in AVM ECs Following Pharmacological Treatment

**Figure 2:**
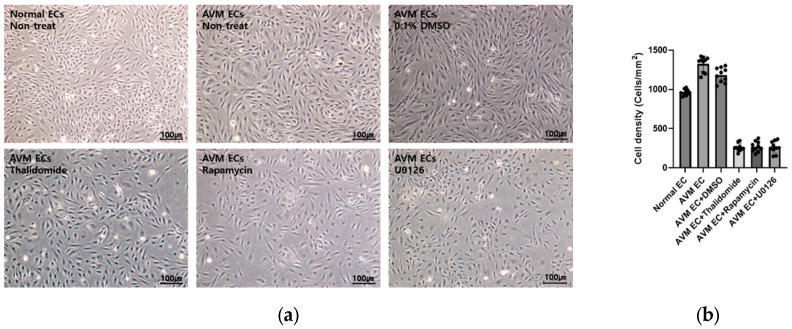
Pharmacological treatment alters cellular morphology and density in AVM ECs. (**a**) Representative microscopic images of ECs after 24 h exposure to pharmacological treatment (×10, scale bar: 100 μm). (**b**) Quantification of ECs density following pharmacological treatment, analyzed using Fiji ImageJ software 1.54h (National Institutes of Health, Bethesda, MD, USA).

Figure 2 presents microscopic images illustrating morphological alterations in AVM ECs after treatment with thalidomide (10 μM, 24 h), rapamycin (10 nM, 24 h), and U0126 (10 μM, 24 h). Compared to untreated normal ECs and AVM ECs, the drug-treated cells exhibited reduced cell density and altered cellular morphology, indicative of impaired angiogenic activity. The thalidomide and rapamycin treatments reduced cell density and minor morphological changes; in comparison, U0126 treatment promoted extensive cellular disintegration. DMSO-treated AVM ECs showed minimal morphological changes compared to the untreated control, indicating that the observed effects were specific to the pharmacological treatments.

#### 3.1.2. Immunofluorescence Images of AVM ECs Treated with Thalidomide, Rapamycin, and U0126

**Figure 3:**
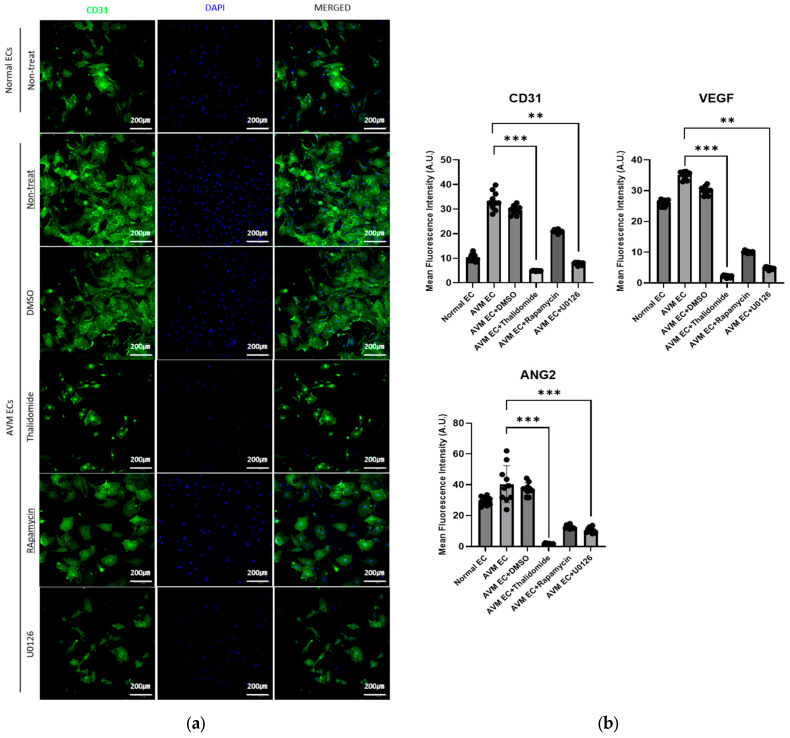

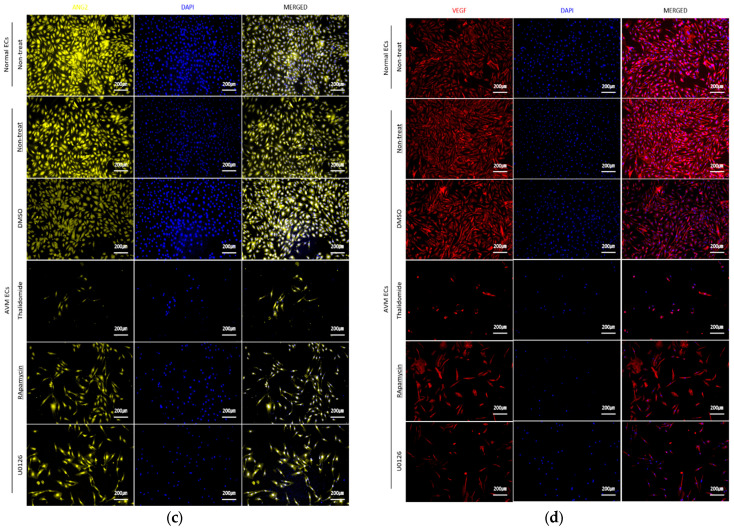
Immunofluorescence image of AVM ECs treated with thalidomide (10 μM, 24 h), rapamycin (10 nM, 24 h), and U0126 (10 μM, 24 h) and stained for CD31 (green), VEGF (red), ANG2 (yellow), and nuclei (DAPI, blue). (**a**) CD31; (**b**) Quantification of fluorescence intensity following pharmacological treatment, analyzed using Fiji ImageJ software 1.54h (National Institutes of Health, Bethesda, MD, USA); (**c**) ANG2; (**d**) VEGF. Scale bar: 200 μm, ** p ≤ 0.01, *** p ≤ 0.001.

Figure 3 presents immunofluorescence images of AVM ECs treated with thalidomide (10 μM, 24 h), rapamycin (10 nM, 24 h), and U0126 (10 μM, 24 h) and stained for CD31 (green), VEGF (red), ANG2 (yellow), and nuclei (DAPI, blue). In comparison with the untreated control, the drug-treated cells exhibited reduced expression and disrupted cytoskeletal organization. The cells treated with thalidomide demonstrated a marked reduction in CD31 and VEGF expression, combined with a decrease in the intensity of Ang2 staining, indicating suppression of endothelial and angiogenic signaling. Rapamycin treatment showed similar but less pronounced effects. The U0126-treated cells exhibited weakened expression of endothelial markers, likely due to the extensive cell lysis and structural disintegration observed after treatment. Quantification of fluorescence intensity confirmed that thalidomide significantly reduced the expression of endothelial markers. DMSO-treated AVM ECs exhibited similar marker expression and morphology to those of the untreated control group, indicating that DMSO had no apparent cytotoxic effect under the treatment conditions.

#### 3.1.3. Expression of Angiogenesis-Associated Genes in AVM ECs After Pharmacological Treatment

**Figure 4:**
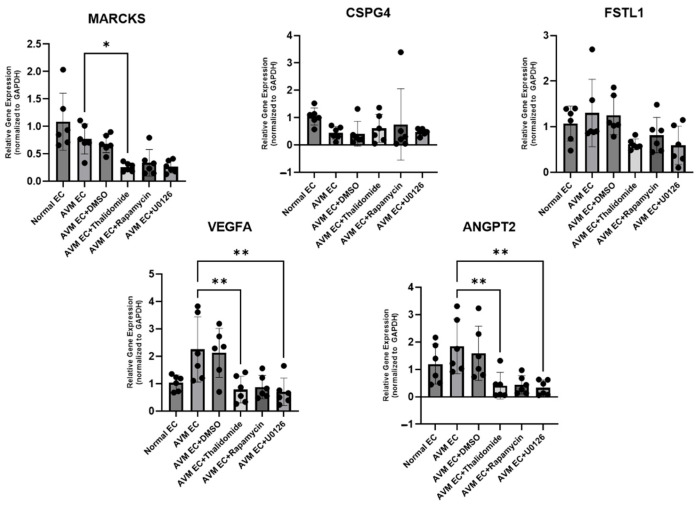
Quantitative real-time PCR analysis of *FSTL1*, *CSPG4*, *MARCKS*, *VEGF*, and *ANG2* expression levels in normal (normal ECs, non-treatment), AVM (AVM ECs, non-treatment), AVM+DMSO (0.1% DMSO-treated AVM ECs), AVM + thalidomide (thalidomide-treated AVM ECs, 10 μM, 24 h), AVM+rapamycin (rapamycin-treated AVM ECs, 10 nM, 24 h), and AVM+U0126 (U0126-treated AVM ECs, 10 μM, 24 h) groups. n = 10; * *p* ≤ 0.05; ** *p* ≤ 0.01.

*FSTL1* expression was evaluated in AVM ECs compared to normal ECs, with a slight decrease observed in the AVM+DMSO group. Pharmacological treatment with thalidomide, rapamycin, and U0126 resulted in a general reduction in *FSTL1* levels. *CSPG3* expression was higher in normal ECs compared to AVM ECs, and treatment with thalidomide and rapamycin partially restored CSPG4 expression in AVM ECs. *MARCKS* expression was lower in AVM ECs compared to normal ECs, and pharmacological treatment did not induce significant changes compared to the untreated AVM groups (Figure 4).

#### 3.1.4. Expression of miR-135b-5p in AVM ECs After Pharmacological Treatment

**Figure 5:**
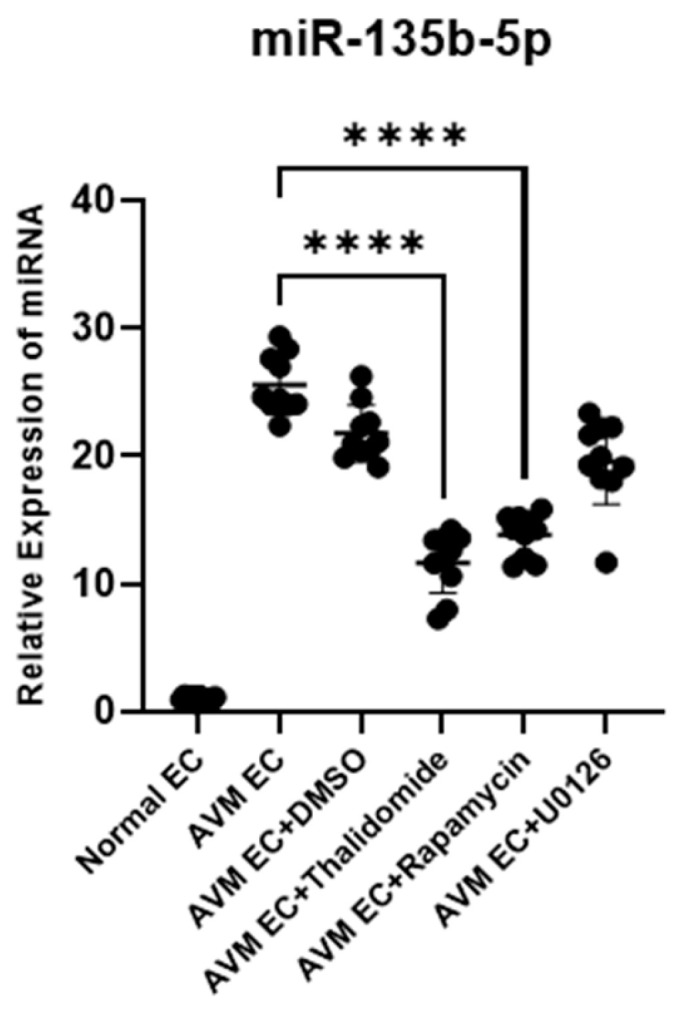
Analysis of miR-135b-5p expression using the TaqMan assay in normal ECs, untreated AVM ECs, DMSO-treated AVM ECs (AVM-DMSO), and AVM ECs treated with thalidomide (10 μM, 24 h), rapamycin (10 nM, 24 h), or U0126 (10 μM, 24 h). Untreated AVM ECs exhibited significantly increased expression of miR-135b-5p compared to normal ECs. Pharmacological treatment resulted in a marked reduction in miR-135b-5p levels, with thalidomide exhibiting the most pronounced effect. Scatter plots represent individual sample values (n = 10 per group), with horizontal bars indicating the mean expression levels (**** *p* < 0.0001).

TaqMan qPCR analysis revealed that miR-135b-5p expression was significantly upregulated in untreated AVM ECs compared to normal ECs (mean fold change ≈ 17.38 vs. 1.049). The DMSO-treated AVM ECs exhibited a slight decrease in miR-135b-5p expression, yet it remained elevated compared to normal levels. Treatment with thalidomide resulted in the most significant reduction in miR-135b-5p levels, followed by a moderate decrease in the U0126 and rapamycin microRNA expression in AVM ECs (Figure 5).

#### 3.1.5. Tube Formation Assay

**Figure 6:**
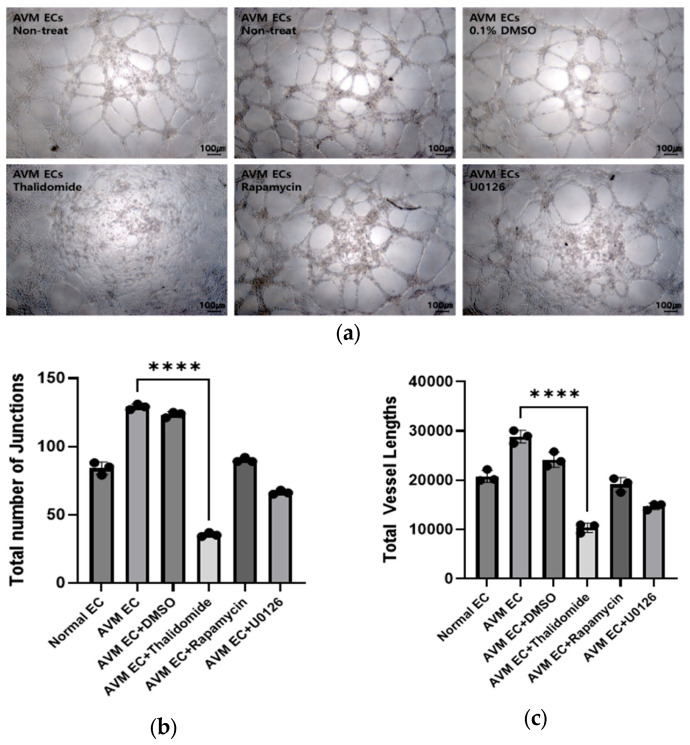
Pharmacological inhibition suppresses tube formation in AVM ECs. Image of AVM EC tube formations: untreated and DMSO-treated controls and those treated with thalidomide (10 μM, 24 h), rapamycin (10 nM, 24 h), or U0126 (10 μM, 24 h), (**a**) Representative images of ECs tube formation assay after 24 h exposure to pharmacological treatment (×10, scale bar: 100 μm). (**b**) Quantification of total number of junctions using Fiji ImageJ software 1.54h (National Institutes of Health, Bethesda, MD, USA), (**c**) Quantification of total vessel lengths using Fiji ImageJ software 1.54h (National Institutes of Health, Bethesda, MD, USA); **** *p* < 0.0001.

Untreated and DMSO-treated AVM ECs formed well-organized capillary-like networks with branching. Thalidomide-treated AVM ECs demonstrated a loss of network formation and exhibited evidence of cellular apoptosis and fragmentation. Rapamycin treatment of AVM ECs reduced network formation, with fewer and thinner structures. U0126-treated AVM ECs failed to form tubular structures, consistent with impaired angiogenic activity (Figure 6).

### 3.2. Immunofluorescent Analysis of AVM Organoids Following Pharmacological Treatment

**Figure 7:**
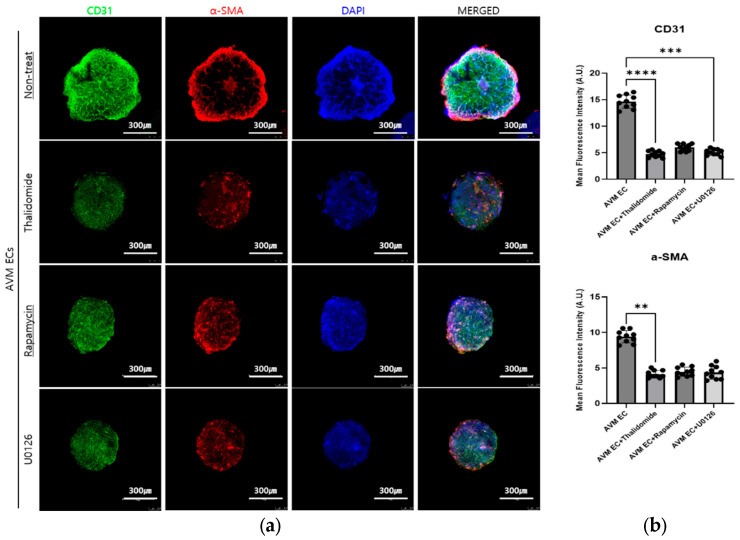
Pharmacological treatment reduces the expression of CD31 (green), α-SMA (red), and DAPI (blue) in AVM blood vessel organoids. Scale bar: 300 μm, ** p ≤ 0.01, *** *p* ≤ 0.001, **** *p* ≤ 0.0001. Whole-mount immunofluorescence image of the AVM blood vessel organoids (BVOs) treated with thalidomide (10 μM, 24 h), and rapamycin (10 nM, 24 h), U0126 (10 μM, 24 h), and stained with CD31 (green), α-SMA (red), and DAPI (blue), (**a**) Representative confocal z-stack images of AVM BVOs, (**b**) Quantification of fluorescence intensity.

The pharmacological treatments resulted in a significant reduction in the expression of vascular smooth muscle markers α-SMA and CD31. Among the treated groups, thalidomide treatment demonstrated the most pronounced suppression of endothelial marker expression and network integrity, suggesting a strong anti-angiogenic effect. The increased nuclear condensation and fragmentation observed in DAPI staining further indicate the induction of apoptosis and cell death in the treated organoids. Quantitative analysis of the CD31-positive area further confirmed a significant decrease in endothelial coverage in the drug-treated BVOs, with thalidomide showing the greatest inhibitory effect (Figure 7).

## 4. Discussion

Despite the clinical challenges associated with treating AVMs, the limited mechanistic understanding continues to hinder the development of effective therapies. Moreover, currently available interventions are largely reactive, often involving invasive procedures with significant risks and variable outcomes. Indeed, although pharmacological agents such as sirolimus and MEK inhibitors have entered clinical trials, the precise mechanisms of action through which these agents affect AVM pathology remain unclear, and their therapeutic efficacy is inconsistent. Furthermore, the lack of standardized disease models for AVMs has posed a significant barrier to both mechanistic studies and the development of drugs.

Recent research on AVMs has led to the development of diverse experimental platforms and animal models, aiming to improve the understanding of their complex genetic and molecular pathogenesis. In vitro systems, such as microfluidic AVM-on-a-chip platforms, have been designed to replicate key pathological features, including vessel dilation and increased permeability [15]. In addition, disease models utilizing mutant mouse models that introduce KRAS mutations (G12V and G12C) into endothelial cells are being employed [16]. Interestingly, beyond genetically modified models such as mice and zebrafish, antibody-based and surgical models are also utilized to mimic AVM pathophysiology [17]. These models are instrumental in drug research targeting pathways activated in AVMs, such as the PI3K/AKT/mTOR and RAS/MAPK/ERK pathways, facilitating a cross-walk strategy for comprehensive AVM treatment [18]. Furthermore, drug approaches that involve the use of existing anti-cancer agents are actively being explored to expand the repertoire of candidate therapies for AVMs, with promising potential for future personalized treatment strategies.

In our study, we utilized EC cultures and AVM organoids to assess the impact of multiple pharmacological agents targeting distinct angiogenic and inflammatory pathways.

The pharmacological treatment concentrations used in this study were selected based on previous studies demonstrating efficacy in endothelial or angiogenic models without evidence of cytotoxicity. The concentrations of U0126 (10 μM, 24 h) were chosen based on the results of previous reports demonstrating its ability to induce or modulate apoptosis in ECs while simultaneously minimizing non-specific toxicity [19]. Rapamycin (10 nM, 24 h) has been shown to suppress EC proliferation and differentiation, downregulate *VEGF* expression, induce apoptosis and autophagy, and promote the cell cycle [20]. Thalidomide (10 μM, 24 h) has been reported to inhibit angiogenesis through the downregulation of *VEGF* and *ANG*, thereby significantly reducing their expression. In addition, thalidomide has been found to effectively inhibit endothelial migration and angiogenic activity [9].

To better understand the molecular mechanisms underlying aberrant angiogenesis in AVMs, we investigated the expression of several genes known to be associated with vascular development and remodeling, including *VEGF*, *MARCKS*, *CSPG4*, *FSTL1*, and *ANG2*. Vascular endothelial growth factor (*VEGF*) is a key mitogen that stimulates the proliferation and migration of ECs, combined with the formation of new blood vessels. Moreover, *VEGF* activates *VEGFR-2* signaling and is widely recognized as the central driver of pathological and physiological angiogenesis [21]. The results of previous studies have demonstrated the role of *VEGF* signaling and hemodynamic stress in AVM formation; however, further in-depth investigation is required to elucidate how distinct molecular pathways contribute to the disease [22]. Angiopoietin-2 (ANG2) modulates vascular remodeling and destabilization. Under pathological conditions, *ANG2* often works synergistically with VEGF to promote angiogenesis by enhancing endothelial permeability and promoting sprouting angiogenesis [23]. Follistatin-like 1 (*FSTL1*) has been reported to play dual roles in angiogenesis, acting as either a pro-angiogenic or anti-angiogenic factor depending on the biological context. *FSTL1* modulates the survival and vascular remodeling of ECs by influencing the *BMP* and *TGF-β* signaling pathways. In addition, *FSTL1* has been implicated in the regulation of cellular proliferation, differentiation, and apoptosis in various pathological conditions [24]. The results of recent studies have demonstrated that *MARCKS* is associated with angiotensin Ⅱ signaling in kidney cancer and plays a critical role in neo-angiogenesis. In one study, a marked reduction in microvessel density was observed in *MARCKS*-knockdown xenograft tumors, as indicated by decreased CD31 expression [25]. Chondroitin sulfate proteoglycan 4 (*CSPG4*) is expressed on pericytes and vascular smooth muscle cells, contributing to vascular stabilization. *CSPG4* plays a role in maintaining the integrity of blood vessels and may regulate tumor angiogenesis by interacting with *VEGFA* pathways [26].

Immunofluorescence analysis was performed to compare the expression of CD31, VEGF, and ANG2. AVM ECs exhibited higher expression of CD31, VEGF, and ANG2 than normal ECs, consistent with their increased angiogenic activity. Pharmacological treatments resulted in a notable reduction in all markers, with the most significant decreases observed in thalidomide-treated groups, wherein extensive apoptosis and suppression of angiogenesis were evident (Figure 3). Quantitative real-time PCR analysis was conducted to assess the impact of pharmacological treatment on angiogenesis-associated gene expression in AVM ECs, revealing that the treatments exert anti-angiogenic effects through the suppression of key molecular aspects in vascular development. *FSTL1* expression was significantly higher in AVM ECs compared to normal ECs. Notably, DMSO treatment did not induce a marked change relative to the untreated AVM ECs. In contrast, treatment with thalidomide (10 μM, 24 h), rapamycin (10 nM, 24 h), and U0126 (10 μM, 24 h) led to a marked reduction in *FSTL1* expression. *CSPG4* expression was notably higher in normal ECs than in AVM ECs. Pharmacological treatment with thalidomide and rapamycin promoted a partial recovery in *CSPG4* expression, suggesting a potential restoration of vascular stability mechanisms. *MARCKS* expression remained lower in AVM ECs compared to normal ECs, with minimal changes observed across the pharmacologically treated groups (Figure 4). The expression of miR-135b-5p, a microRNA biomarker elevated in AVM ECs compared to normal ECs, was significantly reduced following pharmacological treatment compared to normal ECs [27]. Notably, thalidomide treatment resulted in the most pronounced decrease, promoting expression levels closer to those observed in normal ECs. These findings support the regulatory effect of thalidomide on angiogenesis-associated microRNAs (Figure 5). Tube formation assays revealed that AVM ECs exhibited significantly enhanced angiogenic activity compared to normal ECs, forming dense and extensive vascular networks. This finding is consistent with the hyperproliferative and aberrant angiogenic phenotype that is characteristic of AVM pathology. A significant disruption in network formation was observed upon pharmacological intervention, particularly in AVM ECs treated with thalidomide, rapamycin, and U0126, whereby the majority of cells underwent morphological collapse and cell death. These results indicate that targeted pharmacological strategies can effectively suppress the angiogenic potential of AVM ECs. This observation highlights the potential of drug-based approaches for regulating pathological vascular remodeling in AVMs (Figure 6).

Building on our previous development of using AVM organoids as an in vitro model, the pharmacological treatment identified as effective in AVM ECs was applied to the organoid system to evaluate its therapeutic effects. Furthermore, immunofluorescence analysis of AVM organoids, which have been established as an in vitro model, treated with thalidomide (10 μM, 24 h), rapamycin (10 nM, 24 h), and U0126 (10 μM, 24 h) revealed a decrease in the expression of the endothelial biomarker CD31 (green) and the vascular smooth muscle marker α-SMA (red) compared to non-treated AVM organoids. The pharmacologically treated groups show reduced endothelial proliferation and mural cell integrity, in addition to the potential induction of apoptosis, as evidenced by increased nuclear condensation and fragmentation in DAPI-stained cells. Among the treatment groups, thalidomide demonstrated the greatest impact in suppressing CD31 and α-SMA expression, in addition to the most substantial disruption to vascular network integrity. In addition, quantitative analysis of CD31-positive areas provided evidence of a significant decrease in endothelial coverage in the treated groups, supporting a strong anti-angiogenic effect, particularly in the thalidomide groups.

## 5. Conclusions

In this study, we investigated the therapeutic potential of thalidomide, rapamycin, and U0126 in AVMs using patient-derived ECs and organoid models. Among them, thalidomide demonstrated the most significant efficacy by suppressing endothelial proliferation, reducing *VEGF* expression, and downregulating angiogenesis-related miR-135b-5p. Thalidomide also promoted vascular normalization in both two-dimensional (2D; AVM ECs) and three-dimensional (3D; AVM organoids) models, highlighting its promise as a leading candidate for AVM therapy. In addition, the establishment of AVM organoids enabled physiologically relevant in vitro modeling of AVM pathology, providing a useful platform for drug screening and mechanistic studies. While U0126 and rapamycin exhibited complementary pathway-specific effects, the dual anti-angiogenic and anti-inflammatory effect of thalidomide was particularly prominent. In future work, we will focus on refining the AVM organoid system using single-cell analysis to accelerate the development of personalized treatment strategies.

## Figures and Tables

**Table 1:** Characteristics of patient-derived tissue samples of three cell types: normal ECs, AVM ECs, and AVM human dermal fibroblasts for hiPSC reprogramming. Abbreviations: Ant. = anterior; Rt. = right; Lt. = left.

**No.**	**Age**	**Sex**	**AVM Status**	**Sample Type**	**Location**
1	56	M	None	Blood vessel	Ant. neck
2	47	M	None	Blood vessel	Lt. lower leg
3	29	M	None	Blood vessel	Rt. thigh
4	45	M	None	Blood vessel	Rt. groin
5	26	M	None	Blood vessel	Rt. groin
6	8y3m	F	None	Blood vessel	Rt. groin
7	77	M	None	Blood vessel	Rt. groin
8	51	M	None	Blood vessel	Lt. thigh
9	27	M	None	Blood vessel	Lt. trunk
10	72	M	None	Blood vessel	Lt. face
**No.**	**Age**	**Sex**	**AVM Status**	**Sample Type**	**Location**
1	56	M	AVM	Blood vessel	Lt. face
2	7y3m	F	AVM	Blood vessel	Rt. ear
3	6y11m	F	AVM	Blood vessel	Upper back
4	27	F	AVM	Blood vessel	Rt. ear
5	66	M	AVM	Blood vessel	Rt. thumb
6	52	M	AVM	Blood vessel	Rt. glabella
7	29	M	AVM	Blood vessel	Rt. heel
8	23	F	AVM	Blood vessel	Lt. face
9	49	M	AVM	Blood vessel	Lt. forearm
10	55	M	AVM	Blood vessel	Rt. wrist
**No.**	**Age**	**Sex**	**AVM Status**	**Sample Type**	**Location**
1	6y4m	F	AVM	Skin	Lt. face
2	7y3m	F	AVM	Skin	Rt. retroauricular
3	5y11m	M	AVM	Skin	Rt. forearm
4	59	F	AVM	Skin	Lt. groin
5	23	F	AVM	Skin	Lt. face
6	29	M	AVM	Skin	Rt. thigh
7	44	F	AVM	Skin	Rt. ear
8	66	M	AVM	Skin	Rt. groin
9	52	M	AVM	Skin	Rt. glabella
10	49	M	AVM	Skin	Lt. forearm

**Table 2:** Primer sequence used for real-time PCR.

Primer Sequence		
*GAPDH*	Forward sequence	GGAAGGTGAAGGTCGGAGTCA
	Reverse sequence	GTCATTGATGGCAACAATATCCACT
*FSTL1*	Forward sequence	TCGCATCATCCAGTGGCTGGAA
	Reverse sequence	TCACTGGAGTCCAGGCGAGAAT
*MARCKS*	Forward sequence	CTCCTCGACTTCTTCGCCCAAG
	Reverse sequence	TCTTGAAGGAGAAGCCGCTCAG
*CSPG4*	Forward sequence	GTCCTGCCTGTCAATGACCAAC
	Reverse sequence	CGATGGTGTAGACCAGATCCTC
*ANG2*	Forward sequence	TGGCTAGTGACCCCCTACAG
	Reverse sequence	GCTGTGTTCTCTCCAGGCAT
*VEGF*	Forward sequence	GAGTTGCACAGGGGAGGTAT
	Reverse sequence	AGAGGTTAGTGACCCAGCCA

**Table 3:** TaqMan primer/probe sequence for RT-qPCR.

Title	Mature Mirna Sequence (5′-3′)
Endogenous (has-miR-361-5p;)	UUAUCAGAAUCUCCAGGGGUAC
hsa-miR-135b-5p	UAUGGCUUUUCAUUCCUAUGUGA

## Data Availability

The data presented in this study are available within the manuscript.
